# Effectiveness and pulmonary complications of perioperative laryngeal mask airway used in elderly patients (POLMA-EP trial): study protocol for a randomized controlled trial

**DOI:** 10.1186/s13063-019-3351-2

**Published:** 2019-05-08

**Authors:** Ling Zhu, Xiao Shi, Suqing Yin, Jiemin Yin, Ziyu Zhu, Xiong Gao, Yingfu Jiao, Weifeng Yu, Liqun Yang

**Affiliations:** Department of Anesthesiology, Ren Ji Hospital, Shanghai Jiao Tong University School of Medicine, No. 160 Pujian Road, Shanghai, 200127 China

**Keywords:** Laryngeal mask airway, Ventilation mode, Postoperative complication, Elderly patient

## Abstract

**Background:**

With the increasing amount of geriatric surgery, it has become a great challenge for anesthesiologists to reduce the incidence of postoperative pulmonary complications (PPCs). The two most popular airway management methods, laryngeal mask airway (LMA) and endotracheal intubation (ETI), both have their unique advantages in specific clinical settings. For the purpose of helping clinicians make better decisions on airway management during geriatric surgery, we designed this multi-center clinical trial to compare the influence of LMA and ETI on PPCs.

**Methods/design:**

In this multi-center, randomized, parallel clinical trial, a total of 6000 elderly patients, aged ≥ 70 years, with an American Society of Anesthesiologists classification level of 1–2 and a body mass index ≤ 35 kg/m^2^, undergoing elective surgery will be enrolled and randomized into the LMA or the ETI group. Both groups will receive usual perioperative care except for the adoption of LMA/ETI. Primary outcomes are the occurrence of PPCs and patients’ perioperative mortality rates. Ease of intubation, anesthetics consumption, treatment for PPCs, duration of surgery, anesthesia recovery time and performance, time of PPC onset, postanesthesia care unit stay, intensive care unit admission and stay, in-hospital days, re-admission rates, hospitalization cost, and patients’ satisfactory scores will be secondary outcomes. Follow-up will be conducted through phone-call visits until 12 weeks after discharge.

**Discussion:**

This trial will assess the possible benefits or disadvantages of perioperative LMA use in elderly patients compared with ETI regarding the occurrence of PPCs and clinical prognosis. We expect that this trial will also add to the current understanding of PPCs in geriatric populations and contribute to the international recommendations of geriatric surgery management.

**Trial registration:**

ClinicalTrials.gov, NCT02240901. Registered on 16 September 2014.

**Electronic supplementary material:**

The online version of this article (10.1186/s13063-019-3351-2) contains supplementary material, which is available to authorized users.

## Background

Aging is an astonishing demographic transformation faced by the modern world. According to the United Nations, the number of elderly persons will triple over the next 50 years [1]. In China, life expectancy is now > 75 years [2], and by 2050, there will be 100 million people aged older than 80 years [3]. Undoubtedly, along with the increased longevity, the incidence of geriatric surgery will greatly increase, thus posing more challenges in perioperative care for clinical practitioners.

The aging population is a frail group because aging is associated with degenerative changes in anatomical structure, reduced functional tolerance, altered pharmacokinetics, and a higher incidence of morbidity and mortality [4–6]. Cardiovascular, pulmonary, and neurologic complications are the three most common postoperative complications in this population [5]. A postoperative pulmonary complication (PPC), although its definition is still in inadequate consensus, is generally accepted as a clinical measure that encompasses a wide range of postsurgical respiratory outcomes, including pneumonia, atelectasis, and systemic inflammatory response syndrome (SIRS). The incidence of PPCs may vary in different surgery types, with a reported incidence ranging from 1 to 23% [7].

Among numerous risk factors for the development of PPCs, aging is a well-accepted and independent one [7, 8]. Even when the patient is free from respiratory co-morbidity and does not show any evident physical decline before surgery, PPCs can still be triggered by an episode of severe incidence such as surgery and anesthesia [7]. Multiple studies have suggested that patients aged > 60 or 65 years are at higher risk of developing PPCs, and the risk increases with increased age [9, 10]. Other risk factors for developing PPCs include smoking, obesity, pulmonary co-morbidities, positive cough test, prolonged surgery time, limited laryngeal height, and extended forced expiratory time [11, 12].

Geriatric patients often have limited mouth opening and cervical movement, poor cardiovascular tolerance, and altered pharmacokinetics. Under these circumstances [5], a laryngeal mask airway (LMA) is considered a good alternative to endotracheal intubation (ETI) because of its advantageous characteristics such as ease of insertion [13], less irritation to the airway, less reliance on muscle relaxant, and better patient comfort [14]. As a supraglottic airway device, however, it does not separate the trachea from the esophagus; therefore, the LMA is associated with higher incidence of aspiration of gastric contents, especially in susceptible patients with a history of gastroesophageal reflux disease [15]. Moreover, the LMA is not recommended for use in lengthy surgery, morbidly obese patients, or certain other types of surgeries in which more control of the breathing (e.g., heart, lung, and brain surgeries) [16] is required. Therefore, the LMA and ETI both have major roles in daily anesthetic practice, and their influences on PPCs under various circumstances are inevitable topics for question.

In contrast to the popularity of LMA or ETI usage in geriatric surgeries, research evidence about their effect on PPCs is scarce and incomprehensive. Our group published a retrospective single-center study that compared LMA and ETI use in geriatric patients who underwent elective abdominal surgery, and we found that, among those who were transferred into the intensive care unit (ICU), the LMA group was associated with lower incidence of atelectasis and pulmonary embolism [17]. To better clarify whether the use of LMA is superior to ETI for elderly surgical patients and provide sufficient evidence for clinical decision-making, we have designed the current large-scale, randomized, controlled clinical trial. Its rationale, design, and protocol will be described in this paper.

## Methods/design

### Study settings

**Fig. 1:**
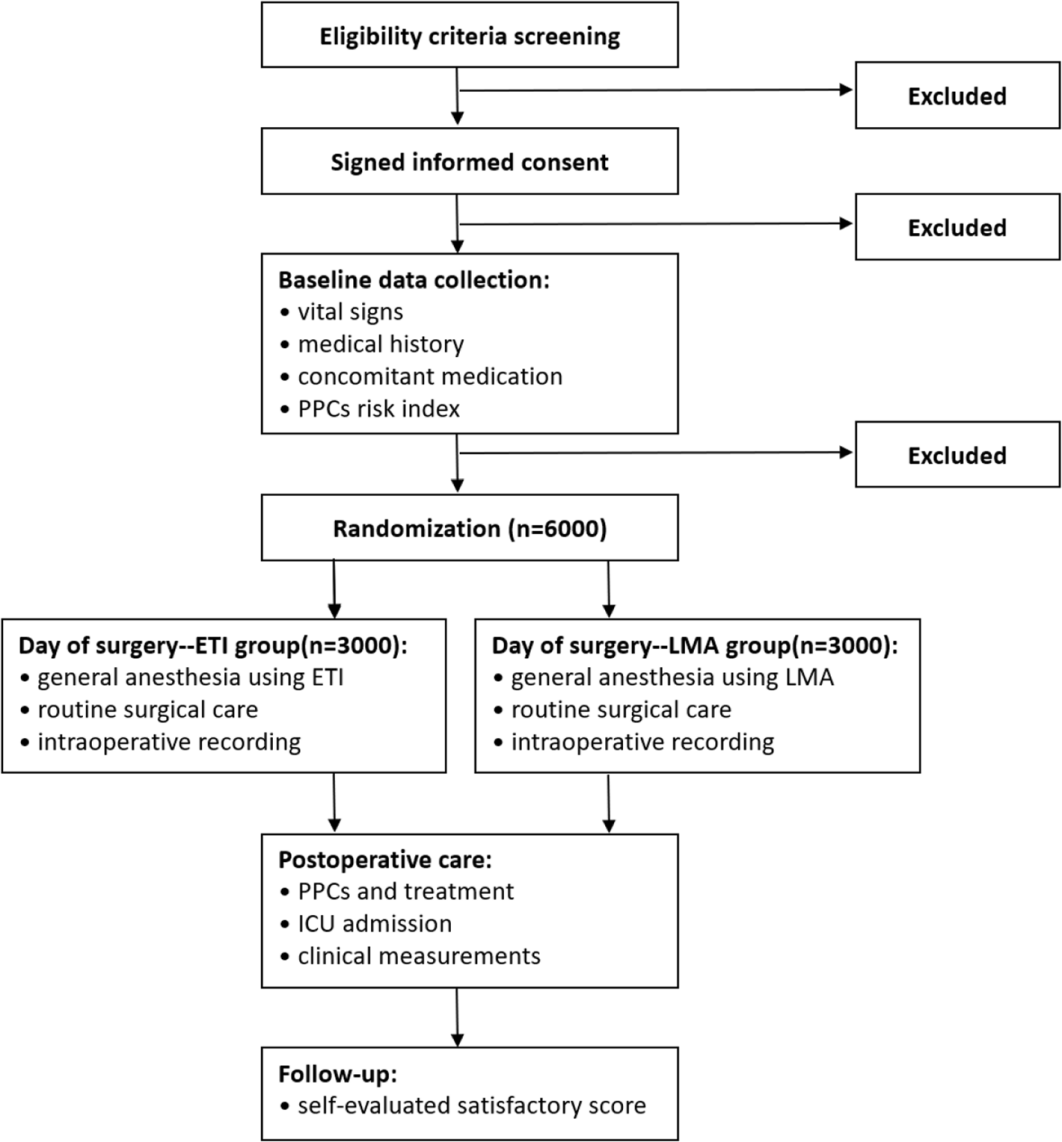
Study flow diagram

The effectiveness and pulmonary complications of perioperative LMA used in elderly patients (POLMA-EP) study is an ongoing, multi-center, randomized, controlled clinical trial with the aim of comparing the effects of the LMA and ETI on PPCs in elderly patients. This trial is registered at ClinicalTrial.gov with ID number NCT02240901. The trial flow chart is shown in Fig. 1. This protocol is in accordance with the Standard Protocol Items: Recommendations for Interventional Trials (SPIRIT) guidelines (see Additional file 1).

We plan to recruit more than 6000 patients from 17 clinical centers in China. The participating clinical centers are listed in Table 1.

**Table 1:** POLMA-EP research clinical centers

Site ID	Site name
1	Ren Ji Hospital (East), affiliated to Shanghai Jiao Tong University School of Medicine
2	Fudan University Shanghai Cancer Center
3	Shanghai Changzheng Hospital
4	Shanghai First Maternity and Infant Hospital
5	Huadong Hospital, Fudan University
6	West China Hospital, Sichuan University
7	Shanghai Guanghua Hospital of Integrated Traditional Chinese and Western Medicine
8	Shanghai Jiading District Central Hospital
9	Shanghai Pudong District Central Hospital
10	Shanghai Huangpu District Central Hospital
11	Shanghai Fengxian District Central Hospital
12	Ren Ji Hospital (West), affiliated to Shanghai Jiao Tong University School of Medicine
13	People’s Hospital of Pudong New District, Shanghai
14	Ren Ji Hospital (South), affiliated to Shanghai Jiao Tong University School of Medicine
15	First Affiliated Hospital of Wenzhou Medical University, Zhejiang Province
16	The Second People’s Hospital of Wuxi, Jiangsu Province
17	First Affiliated Hospital of Xiamen University, School of Medicine

### Inclusion and exclusion criteria

The inclusion criteria are as follows: (1) age ≥ 70 years, (2) elective surgery, (3) American Society of Anesthesiologists (ASA) classification I–II, (4) body mass index (BMI) ≤ 35 kg/m^2^, (5) provision of signed informed consent.

The exclusion criteria include patients who (1) require emergency surgery; (2) have anticipated difficult intubation; (3) have a broken or unstable cervix; (4) have laryngeal disease; (5) are at high risk of aspiration (gastroesophageal reflux disease, full stomach); (6) are unable to cooperate for any reason, such as inability to speak or understand, mental disease, or inability to go to the clinics; (7) have taken experimental drugs in the preceding 3 months or joined another clinical trial; (8) did not provide informed consent or have withdrawn consent; (9) are evaluated by the investigators as unsuitable for this trial.

### Ethics issues

This study is approved by the Ethics Committee of Ren Ji Hospital, Shanghai Jiao Tong University School of Medicine. Surgery will be performed within 2 weeks after the screening process. Designated doctors will explain this trial to interested potential participants in detail and provide them with the informed consent form. Participants are given at least 24 h to decide whether they wish to participate in this trial. The informed consent form will be signed by the participant or his/her trustee or guardian and may be withdrawn at any time during the trial. Written informed consent and the patient’s baseline data will be obtained before randomization. Moreover, participants are encouraged to contact the research team if they have any health concerns during the trial.

### Blinding, randomization, and allocation concealment

Given the nature of the intervention, blinding of caregivers or patients is not possible. However, investigators responsible for data collection and statisticians will be blinded to the study arms.

Random numbers and group allocations are generated with a 1:1 ratio and stratified according to PPC risk scores (listed in Table 2) by a central randomization system known as an interactive web response system (IWRS) developed by Beijing Blue Balloons Technology Company Limited (http://www.blueballon.cn). Allocations generated by the IWRS will be stored in opaque and numbered envelopes. Participants will be given a randomization number by the designated anesthesiologist and will be allocated according to indications inside that numbered envelope. If participants decide to withdraw after assignment, their clinical information will not be used in this trial, but their assigned random number will still be retained.

**Table 2:** PPC risk index

Independent risk factors	Risk score
Age > 70 years	1
Cough and sputum production	1
Diabetes	1
Smoker (or patient quit within the past 6 months)	1
COPD	1
BMI > 27 kg/m^2^	1
FEV1 < 80% and FEV1/FVC < 70%	2

*COPD* chronic obstructive pulmonary disease, *BMI* body mass index, *FEV1* forced expiratory volume in 1 s, *FVC* forced vital capacity

In emergent cases when the LMA or the ETI tube cannot be placed successfully after multiple attempts, anesthesiologists may change the ventilation method or administer different treatments according to their best clinical judgement, and the patient will be excluded from the trial.

### Intervention

Apart from the trial airway management method (LMA or ETI), other clinical decisions will be made by the practitioners at each site according to their clinical judgement and hospital protocol, including anesthesia induction, maintenance and recording, anesthetic drug use and dosage, surgical approach, and ICU treatment.

### Primary outcomes

The primary outcomes are the incidence of PPCs (diagnostic criteria shown in Tables 3 and 4) and perioperative mortality rate.

**Table 3:** Classifications of PPCs

Grading	Definition
0	No pulmonary complications
1	Any one of the following: cough, minor lung atelectasis (no obvious cause), dyspnea (no obvious cause)
2	Cough and sputum production (no obvious cause), bronchospasm (new-onset asthma or exacerbation of existing asthma that requires treatment), hypoxemia (SpO2 < 90% or PaO2 < 60 mmHg while breathing air), atelectasis (diagnosed by radiography or abnormal pulmonary sign with T > 37.5), hypercapnia (temporary or requires treatment; caused by medication or over-ventilation)
3	Pleural effusion (that requires drainage), suspected pneumonia (indicated by radiography, with or without microbiologic findings), diagnosed pneumonia (diagnosed based on the combination of radiology finding and microbiology evidence such as Gram staining or culture), pneumothorax (suggested by X-ray: observation of a lucent gas space devoid of pulmonary vessels between visceral pleura and the parietal pleura or symptoms of dyspnea, chest pain, irritating cough), noninvasive or invasive mechanical ventilation < 48 h
4	Respiratory failure (that requires noninvasive or invasive mechanical ventilation for > 48 h)

*PaO2* partial pressure of oxygen, *SpO2* blood oxygen saturation, *T* body temperature

**Table 4:** Definition of PPCs

Complications	Diagnostic criteria
Pneumonia	X-ray finding of a new or progressive pulmonary infiltration and meeting 2 of the following 3 indications:
	(1) Cough, exacerbation of dyspnea or purulent sputum
	(2) Body temperature above 38 °C or under 36 °C
	(3) WBC > 12,000 or < 4000/μL
Atelectasis	Mainly based on X-ray findings as follows:
	Lung collapse
	Compensatory hyperinflation of adjacent ipsilateral lung tissue
	Wedge- or linear-shaped opacities
	Shifting of mediastinum
	Diaphragm move towards the collapse
Pleural effusion	X-ray finding of obscure or disappearance of costophrenic angle, obscured ipsilateral diaphragm
SIRS	(1) Core body temperature > 38 °C or < 36 °C
	(2) HR > 90 bpm, in cases of atrial arrhythmia, ventricular rate > 90 bpm; exclude when used medications that may affect heart rate
	(3) RR > 20 bpm, or PaCO2 < 32 mmHg (4.2 kPa), or urgent mechanical ventilation used
	(4) WBC > 12,000 or < 4000/μL
Sepsis	(1) Positive microbiologic culture from blood, or definite tissue infection, presence of abscess
	(2) Meets at least 2 SIRS definitions
ALI/ARDS	(1) Lung injury of acute onset (within 1 week or progressive)
	(2) Hypoxemia, PaO2/FiO2 ≤ 300/200 mmHg
	(3) X-ray showing obscured bilateral lobes which cannot be explained by effusion, atelectasis, or nodules
	(4) Respiratory failure that cannot be explained by heart failure or hypervolemia

*PaO2* partial pressure of oxygen, *FiO2* fractional inspired oxygen concentration, *HR* heart rate, *RR* respiratory rate, *WBC* white blood cell, *SIRS* systemic inflammatory response syndrome, *ALI* acute lung injury, *ARDS* acute respiratory distress syndrome

### Secondary outcomes

Secondary outcomes include ease of intubation (number of intubation attempts, disturbance to the following gastric tube insertion), anesthetics consumption, treatment for PPCs, duration of surgery, anesthesia recovery time and performance, time of PPC onset, postanesthesia care unit (PACU) stay, ICU admission and stay, re-admission rate, in-hospital days, hospitalization cost, and patients’ satisfactory score.

### Adverse events and serious adverse events

An adverse event (AE) refers to an untoward medical occurrence that happens during the trial. A serious adverse event (SAE) refers to any incident that is life-threatening or may cause significant disability, including but not limited to SIRS, cardiocirculatory instability, stroke, organ failure, or death. All AEs and SAEs will be treated immediately, and the type of event, likely cause, place and date of occurrence, and treatment of the events will be documented. SAEs must be reported to the principal investigator within 24 h and will be discussed in data monitoring committee (DMC) meetings.

### Data collection and follow-up

**Fig. 2:**
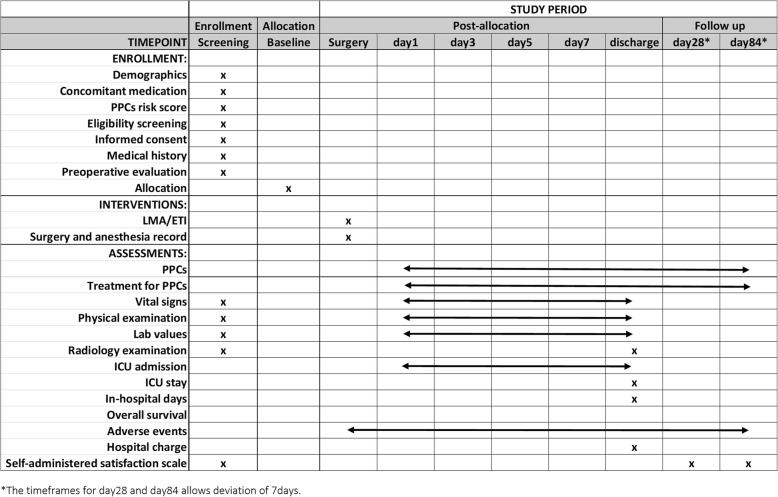
Standard Protocol Items: Recommendations for Interventional Trials (SPIRIT) figure for the POLMA-EP trial

Participants will be evaluated and their medical information will be collected and documented according to Fig. 2.

Upon arrival, a series of baseline information will be collected, including the patient’s demographics, cause of hospitalization, elective surgery type, medical history, anaphylactic history, and co-morbidities and concomitant medication. Physiological parameters will also be collected, including blood pressure (BP), heart rate (HR), respiratory rate (RR), core body temperature (or axillary temperature + 0.5), ASA classification level, and risk for PPCs. The patient’s cardiovascular, neurological, respiratory, endocrine, and urinary functions will be evaluated and recorded. Laboratory tests including routine blood test, blood gas analysis, and pulmonary function indicators such as vital capacity (VC), forced vital capacity (FVC), forced expiratory volume in 1 s (FEV1), and peak expiratory flow (PEF) will also be measured and documented.

On the day of surgery, anesthetic and surgical recording will also be completed. This will include anesthesia type and duration, mechanical ventilation length, surgery time, PACU stay, blood products (red blood cells/plasma/platelets/fresh frozen plasma/albumin) transfusion, fluid infusion (crystalloid solution and artificial colloids), amount of bleeding, intubation type, times of attempts, ventilation mode, positive end-expiratory pressure (PEEP), tidal volume, types and dosage of anesthetic drugs, and ICU admission and stay.

The PPC level, routine blood tests, blood biochemistry, and blood gas analysis of each patient are evaluated at days 1, 3, and 7 and before discharge. Before discharge, important examinations such as pulmonary radiography and sputum and blood cultures will be completed and recorded. For PPCs, treatments including medication (drug name and dose) and mechanical ventilation (mode, duration, and other parameters) will be documented. In cases of death, the time, cause, and relativity to surgery or anesthesia will be evaluated and recorded.

A brief summary of discharge will include discharge diagnosis, hospitalization stay, ICU admission and length, and hospitalization fee. Follow-up will be done on day 28 ± 7 after surgery. Apart from the PPC score, a self-evaluating questionnaire covering overall health, daily activity level, limitations during work, pain, energy, social activity, passive emotions, personal issues, accompanying pulmonary diseases, and overall satisfaction will be completed. Another follow-up will take place on day 84 ± 7 post surgery. The follow-up will be in the form of phone-call visits by designated allocation-blinded clinicians.

Data will be collected on paper case report forms (CRFs) by nurses or clinicians blinded to group allocation and then collected onto electronic CRFs (eCRFs) by trained assistants. The eCRF is a restricted online system that is capable of alerting information inconsistencies, missing data, and atypical errors. The database will be available for editors and peer reviewers when required.

### Data monitoring

The DMC, composed of principal investigators from each center, statisticians, and representatives from the ethics committee, will be responsible for data monitoring. Members of the DMC are independent of the sponsors. Written reports on trial progress and feedback will be submitted to the committee quarterly, and cases of unexpected scenarios and SAEs will be discussed at committee meetings.

## Statistics and data analysis

### Sample size estimations

Power Analysis and Sample Size (PASS) software (version 11.0; Number Cruncher Statistical Software (NCSS), LLC, Kaysville, UT, USA) was used for the sample size calculation. The χ^2^ test for a multiple proportions procedure was used. With an α = 0.05, a power of 90%, an effect size = 0.09, and degrees of freedom = 2, we estimated that 2584 patients would be required for our study. If the attrition rate was set at 10%, a total of 6000 patients (3000 in each group) would be required. The effect size was calculated from the assumption that the LMA would cause a reduction from 15% to 10% for the incidence of PPCs. The incidence of PPCs for patients undergoing different types of surgeries has been reported to be in the range of 1–23% [5]. Therefore, a proportion of 10% of the patients in the LMA group were expected to develop PPCs, and this was used to calculate the sample size.

### Statistical analysis

Statistical analyses will be performed by an independent statistician using SAS 9.4 software (SAS Institute Inc., Cary, NC, USA). After normality testing, the patients’ data will be presented as the mean and standard deviation (SD) if they are normally distributed; otherwise, they will be presented as the mean and interquartile range. To compare numerical data, a paired or an unpaired *t* test will be used for normally distributed data, and the Kruskal-Wallis test will be used for skewed data. The χ^2^ test and Fisher’s exact test will be used to compare proportional data. When two χ^2^ tests are performed, the *P* value is adjusted to 0.025. A *P* value < 0.05 will be considered statistically significant. If *P* < 0.05, the Nemenyi test will be used to compare the difference between two groups. A logistic regression model will be developed to detect any potential confounding factors.

## Discussion

A tremendous amount of geriatric surgeries are performed around the world [18]. For elderly people, PPCs are a prevalent and serious threat [19, 20]. Reducing the risk of PPCs has always been a complex question, and investigators have put great effort into studying this topic. Previous studies have looked into interventions including inspiratory muscle training [21], smoking intervention [12], and respiratory rehabilitation [22, 23] to reduce the incidence of PPCs. Regarding intraoperative management, it was reported that low-tidal-volume ventilation or PEEP is effective in the prevention of PPCs [24, 25]. However, the LAS VEGAS study [26] published in 2017 and the PROVHILO study [27] published in 2014 failed to show the same result. Likewise, studies on high-flow oxygen have also reached obscure conclusions [28, 29]. The use of the LMA was reported to be superior to ETI in prehospital emergency care [30, 31], but its influence on PPCs in surgical elderly people lacks clinical evidence [20]. Therefore, this study is much needed to help optimize intraoperative airway management in this population.

There are several noteworthy limitations of this study. Firstly, all participating centers are located in east China; therefore, one may not postulate that results from this study can be implemented directly to other places or populations. Secondly, the long-term follow-up results are mainly based on patients’ self-evaluated questionnaires, which potentially can add bias to the results of this study. Thirdly, we are unable to apply uniform treatment to all patients. The ventilation mode adopted during anesthesia, administration of analgesics, and the prophylactic use of antibiotics, anticholinergics, and steroids are decided according to clinicians’ best judgement and will be recorded for future subgroup analysis. Other potential confounding factors [20], including surgery type and duration, will also be evaluated during statistical analysis. Moreover, the LMA device has many different forms and configurations. In this study, it is unreasonable to designate a single type of LMA device, but the mode of LMA used will be recorded. Lastly, the patient is not blinded in this trial, because extubation criteria need to be met before the LMA or the ETI tube can be removed. Even though we will make sure that every participant is informed not to reveal his/her group allocation to assessors, there is still the risk of assessor unblinding. In such cases, the patient will be evaluated by another assessor.

With our multi-center, randomized, controlled study, we aim to evaluate the safety profile of LMA/ETI use and add to the knowledge pool of airway management in elderly patients. The findings from this study can be used to help with clinicians’ understanding of the issue and can be used in clinical settings when decisions need to be made.

### Trial status

The trial is currently in patient recruitment.

## Additional file

Additional file 1: SPIRIT 2013 checklist: recommended items to address in a clinical trial protocol and related documents. (DOC 121 kb)

## Abbreviations

AE: Adverse event
ASA: American Society of Anesthesiologists
BMI: Body mass index
BP: Blood pressure
COPD: Chronic obstructive pulmonary disease
CRF: Case report form
eCRF: Electronic case report form
ETI: Endotracheal intubation
FEV1: Forced expiratory volume in 1 s
FVC: Forced vital capacity
HR: Heart rate
ICU: Intensive care unit
IWRS: Interactive web response system
LMA: Laryngeal mask airway
PACU: Postanesthesia care unit
PEEP: Positive end-expiratory pressure
PEF: Peak expiratory flow
PPC: Postoperative pulmonary complication
RR: Respiratory rate
SAE: Serious adverse event
SIRS: Systemic inflammatory response syndrome
VC: Vital capacity
