# Correction to: Effectiveness and pulmonary complications of perioperative laryngeal mask airway used in elderly patients (POLMA-EP trial): study protocol for a randomized controlled trial

**DOI:** 10.1186/s13063-020-04268-4

**Published:** 2020-03-30

**Authors:** Ling Zhu, Xiao Shi, Suqing Yin, Jiemin Yin, Ziyu Zhu, Xiong Gao, Yingfu Jiao, Weifeng Yu, Liqun Yang

**Affiliations:** Department of Anesthesiology, Ren Ji Hospital, Shanghai Jiao Tong University School of Medicine, No. 160 Pujian Road, Shanghai, 200127 China

**Correction to: Trials**

**https://doi.org/10.1186/s13063-019-3351-2**

After publication of our article [1] we have been notified on a mistake in the sample size.

According to the calculation described in the article, sample size should be 2026 in total (*n* = 1013 per group), instead of 6000. Moreover, degrees of freedom should equal to 1, since there are two study groups.

The effect size was calculated from the assumption that the LMA would cause a reduction from 15 to 10% for the incidence of PPCs. We estimated that 1822 patients (911 per group) would be required for our study. If the attrition rate was set at 10%, a total of 2026 patients (1013 in each group) would be required.

Figure 1 has also been adjusted.

**Fig. 1:**
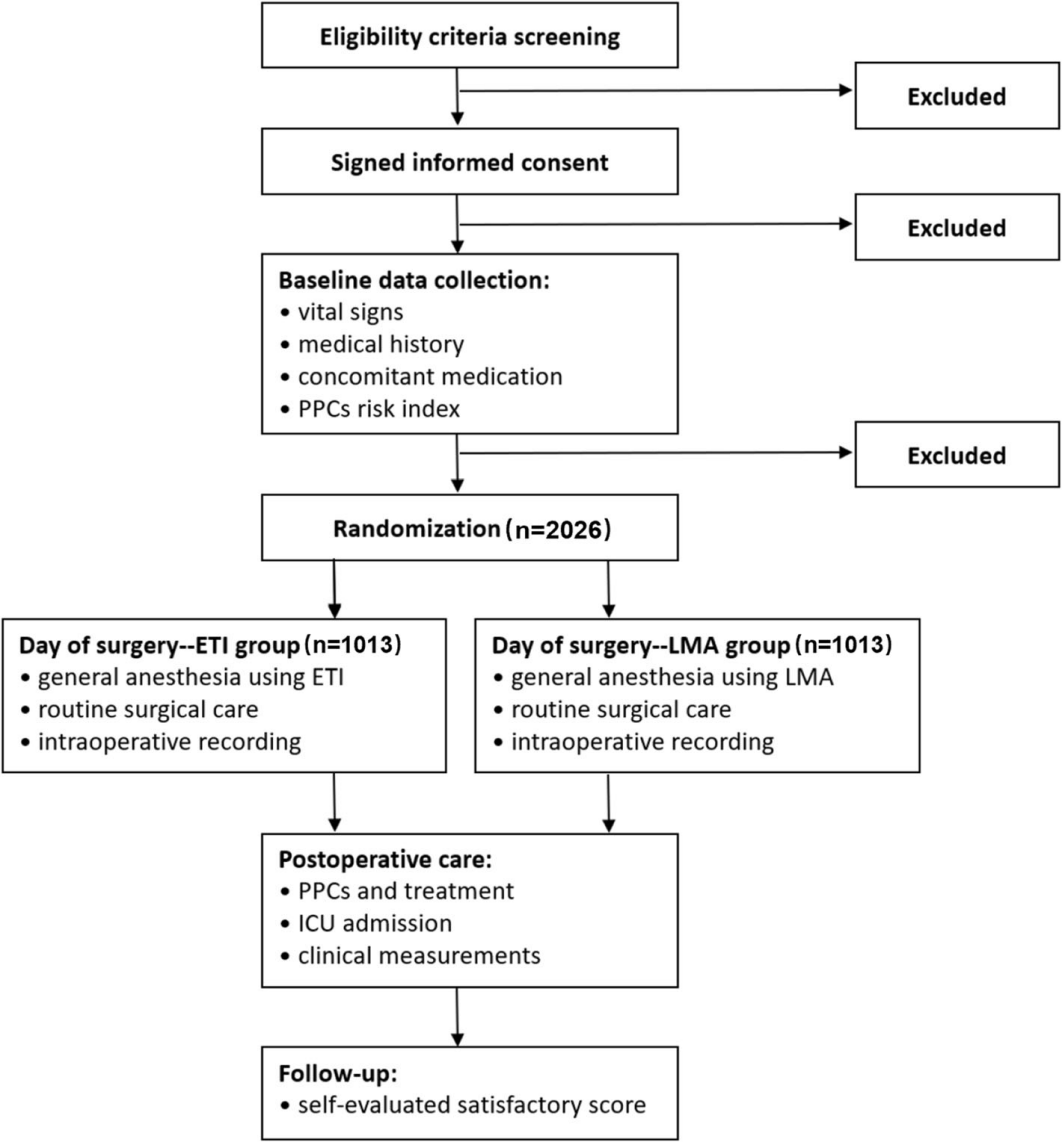
Study flow diagram
